# A panel of four autoantibodies to tumour-associated antigens in patients with prostate cancer and its potential for multi-cancer detection

**DOI:** 10.1038/s41416-025-03242-8

**Published:** 2025-11-18

**Authors:** Cuipeng Qiu, Xiao Wang, Giulio Francia, Carlos A. Casiano, Jian-Ying Zhang

**Affiliations:** 1 Department of Biological Sciences & NIH-Sponsored Border Biomedical Research Center, The University of Texas at El Paso, El Paso, TX USA; 2 Center for Health Disparities and Molecular Medicine, Department of Basic Sciences, Cancer Center, Department of Medicine/Division of Rheumatology, Loma Linda University School of Medicine, Loma Linda, CA USA

**Keywords:** Tumour biomarkers, Tumour immunology, Diagnostic markers

## Abstract

**Background:**

Genomic alterations can drive tumorigenesis, and understanding the immune response to those alterations may aid in developing new targets for diagnosis and therapy. Tumour-associated antigens (TAAs) are self-antigens that are abnormally expressed in tumours. Autoantibodies (AAbs) triggered by TAAs have been considered reporters of early carcinogenesis. This study aimed to profile AAbs to overexpressed or driver gene-related proteins (DRPs) in prostate cancer (PCa).

**Methods:**

Twenty-nine targets including 14 overexpressed proteins and 15 DRPs were screened via serological proteome analysis and bioinformatics analysis, respectively. ELISA was then performed to assess their corresponding AAbs in 293 serum samples. Immunohistochemistry (IHC) was used to determine the tissue expression of TAAs.

**Results:**

Nineteen AAbs showed significantly higher serum levels in PCa patients than in normal controls. A panel with four AAbs (PIK3CA, SPOP, IF4H, HSP60) was developed, showing an AUC of 0.901. A differential AAb response to these four TAAs was observed in three distinct PCa populations. The panel was evaluated across six other common cancers including 445 serum samples, showing potential for multi-cancer detection. High expression of the four TAAs targeted by AAbs was found in PCa tissues by IHC.

**Conclusions:**

These findings suggest that overexpressed proteins or DRPs may have altered immunogenicity, leading to the production of corresponding AAbs.

## Introduction

Prostate cancer (PCa) is the second most frequent cancer in males with 1.47 million new cases and 396,700 deaths worldwide in 2022 [1]. It is the second leading cause of cancer-related deaths among U.S. males with over 35,000 estimated deaths in 2025 [2]. PCa is a type of indolent cancer that if not detected and treated early can progress to an advanced metastatic castration-resistant (mCRPC) stage, which is associated with high mortality [3]. Although recent advances in the treatment of mCRPC using next generation androgen receptor (AR) signalling inhibitors, novel drug combinations, and theranostic approaches have improved overall survival, patients with mCRPC and its most aggressive neuroendocrine form (NEPC) invariably succumb to the disease [4]. Screening precancerous lesions or early tumour detection is an effective strategy to reduce the mortality of cancer [5]. Several blood-based biomarkers have been introduced as noninvasive approaches in the diagnosis, staging, and prognosis of PCa [6]. However, many of these biomarkers are limited by low sensitivity and specificity, which were not optimal enough for the application to early detection. For instance, prostate-specific antigen (PSA) screening can reduce the risk of PCa, but the suboptimal sensitivity and specificity of the PSA test may lead to unnecessary biopsies or overtreatment [7–9]. These limitations led the US Preventive Services Task Force (USPSTF) to make specific recommendations regarding PSA screening for PCa diagnosis [10]. Detecting blood-based biomarkers is an effective screening tool to achieve early cancer detection. Given the limitations of PSA screening, it is imperative to identify novel blood-based biomarkers that could complement PSA for the early detection of PCa with high sensitivity and specificity. This would be of enormous benefit to high-risk groups, including men of African Ancestry and certain Hispanic/Latino subgroups, who have higher PCa incidence and poor survival rates compared to other groups, and for which the USPSTF recommendations regarding PSA screening may not be applicable [11–13].

Antigenic changes during tumorigenesis can be recognised by the immune system of cancer patients resulting in the production of circulating autoantibodies (AAbs) targeting tumour-associated antigens (TAAs) [14, 15]. TAAs are self-proteins that are aberrantly expressed, mutated, or displaying neoepitopes, and can be recognised by the host immune system to generate autoantibodies in cancer patients [14, 16]. Cancer patients with aberrantly expressed proteins, such as p53, often have high levels of circulating anti-p53 autoantibodies that can be detected in their sera [17]. This aberrant expression and ensuing generation of autoantibodies can be further related to p53 gene mutations accumulated in the tumour, which may result in elevated p53 mRNA and protein expression levels [18]. As another example, we previously reported the presence of anti-PDLIM1 AAbs in sera of ovarian cancer patients accompanied by elevated levels of PDLIM1 mRNA and protein expression within ovarian tumour tissues [19]. Furthermore, the development of certain cancers involves mutations in a set of genes called “cancer driver genes” which affect cellular functions [20]. Proteins encoded by these driver genes may play important roles in cancer [20, 21]. Therefore, altered proteins – overexpressed or driver gene-related proteins (DRPs) in tumours might serve as TAAs leading to the production of corresponding AAbs.

Circulating AAbs triggered by TAAs have emerged as promising biomarkers for cancer diagnosis [3, 22, 23]. They are considered reporters of early carcinogenesis and indicators of cancer prognosis [24]. These AAbs are abundant, detectable in early disease stages, and stable in the serum of cancer patients where they can be detected months or even years before the clinical diagnosis of cancer [25]. However, the diagnostic application of individual AAbs is limited by their low sensitivity in certain cancers. An optimised autoantibody panel, on the other hand, could enhance their ability to serve as biomarkers to detect cancers [26]. Also, the detection of multiple AAbs can be accomplished via non-invasive methods using only a small amount of blood-derived serum samples. We hypothesised that altered proteins/TAAs may trigger immune responses in PCa patients to generate circulating AAbs that could be used as blood-based biomarkers to facilitate PCa detection non-invasively. In this study, we used serological proteome analysis (SERPA) and PCa-related driver genes to identify novel AAbs to overexpressed proteins and DRPs in PCa. Bioinformatics was used to screen driver genes with high mutations and enrichment in PCa based on published databases [20, 21, 27]. Human samples including cell lines (PC3, 22RV1, LNCaP), and patient sera and tissues were also used in this study to comprehensively profile PCa patient serum AAbs targeting overexpressed proteins or DRPs. We benchmarked different classification algorithms (logistic regression, SVM, random forest) to develop an optimal AAb panel with high diagnostic performance in PCa. The panel was tested in serial sera from PCa patients before and after surgery to explore its correlation with surgery. In addition, the panel was evaluated across six common cancers including lung cancer (LC), hepatocellular carcinoma (HCC), gastric cancer (GC), colorectal cancer (CRC), breast cancer (BC), and cervical cancer (CC). We also validated TAA expression in PCa tissue microarray to confirm the consistency of protein expression in antigen-autoantibody reactions. This study identified a panel of AAbs targeting TAAs associated with DRPs in PCa that could serve as potential biomarkers for early cancer detection and management.

## Materials and methods

### Serum samples and cell lines

A total of 773 serum samples were analysed in this study, which included 203 sera from patients with PCa, 35 serial sera collected from 13 PCa patients before and after surgery, 90 sera from normal controls (NC), and 445 sera from six common cancers (80 LC, 81 HCC, 77 GC, 81 CRC, 81 BC, and 45 CC). Among the PCa patients, 59 patients (average age 70 years) self-identified as African American (AA), 100 patients (average age 67 years) self-identified as European American (EA) were originally obtained from Loma Linda University (LLU) Medical Centre (CA, USA). Serum samples derived from 21 PCa patients (average age 68 years) who self-identified as Hispanic American (HA), PCa patients before and after surgery, NC, and common cancers were obtained from the serum bank in the Cancer Autoimmunity and Epidemiology Research Laboratory of the University of Texas at El Paso (UTEP, TX, USA). PSA levels of 35 serial sera from 13 PCa patients (average age 60 years) were available. There were 5 of 13 patients (38.5%) who had abnormal PSA levels ( ≥4 ng/mL) before surgery (radical prostatectomy). Informed consent has been obtained from all patients. This study was approved by both the Institutional Review Boards of UTEP and LLU.

Three human PCa cell lines PC3 (CRL-1435, ATCC), 22Rv1 (CRL-2505, ATCC), and LNCaP (CRL-1740, ATCC) were cultured in RPMI-1640 Medium (GIBCO, Life Technologies, USA) with 10% fetal bovine serum, 100 U/ml penicillin and 100 U/ml streptomycin in a humidified incubator (37 °C, 5% CO_2_) and maintained according to the ATCC handling procedure. Cells were collected when they grew to 90.0% confluent in a T75 tissue culture flask (FALCON, USA) for SERPA.

### SERPA

SERPA was performed as previously described [28]. First, two-dimensional polyacrylamide gel electrophoresis (2-DE) was performed to separate whole proteins from the individual PCa cell lines. In brief, whole cell lysates of PC3, 22Rv1, and LNCaP were prepared by lysis in rehydration buffer (Bio-Rad, USA). Whole protein concentrations were quantified by Bradford assay (Bio-Rad, USA) and incubated with 7 cm immobilised pH gradient (IPG) strips (200 µg/strip), pH 3-7 (Bio-Rad, USA). IPG strips were subjected to isoelectric focusing (IEF) first dimension separation at 250 V for 30 min, 4000 V for 1.5 h, and 4000 V for 5 h to 25,000 V-h. The focused IEF strips were then equilibrated with equilibration buffer (Bio-Rad, USA) and placed into SDS-PAGE gel to run the second-dimension separation. For each cell line, three gels were run simultaneously, in which the first one was used to probe PCa sera, the second to probe NC sera, and the third to visualise protein spots by staining with Coomassie blue R-250. Western blotting (WB) was conducted to detect immunoreactivity of sera from PCa and NC against protein spots. Briefly, after the second-dimension separation, proteins from 2-DE gels were transferred to nitrocellulose membranes and incubated with either pooled sera from PCa or pooled sera from NC (1:200 dilution) overnight at 4 °C. The pooled PCa sera were previously screened via WB for positive immunoreactions to whole proteins extracted from PCa cell lines. In contrast, pooled sera from NC showed negative immunoreactions to these proteins. Horse-radish peroxidase (HRP)-conjugated goat anti-human IgG (Invitrogen, USA) was used as a secondary antibody at 1:5000 dilution and incubated with membranes for 1 h at room temperature. Immunoreactive spots were detected by WesternSure PREMIUM Chemiluminescent Substrate (LICORbio, USA). Finally, the immunoreactive spots of interest from three PCa cell lines were analysed by mass spectrometry (MS + MS/MS), which was conducted by Applied Biomics (Hayward, USA). The detailed procedures for in-gel digestion of protein spots and MS were described in a previous study [28].

### Driver genes screening

Three strategies were used to screen PCa-related driver genes: (1) cancer driver genes datasets (accessed in May 2022) of nine cohorts containing 1503 samples from the IntOGen database [20] for primary PCa were analysed. We filtered 63 driver genes in PCa and the top 20 driver genes with high mutations and high frequencies in the nine cohorts were screened. (2) The Kyoto Encyclopaedia of Genes and Genomes (KEGG) pathway analysis was performed on the 63 genes selected in previous step, and 16 driver genes that were enriched in PCa pathways were obtained. (3) Based on published research which reported driver genes across 28 tumour types [21], we selected the top 17 driver genes that were highly related to PCa. Of these, 15 driver genes that appeared in at least two screening strategies were selected. The enrichment analysis of the screened genes was performed by STRING (string-db.org).

### ELISA

We conducted the enzyme-linked immunosorbent assay (ELISA) to test the serum levels of AAbs to candidate TAAs. The detailed procedures were described previously [23, 29]. A total of 29 recombinant proteins were used to probe the corresponding AAbs. Detailed information on these recombinant proteins is listed in Table S1. Quality of proteins were confirmed by SDS gels (Fig. S1). Proteins were diluted and coated onto 96-well plates overnight at 4 °C, and serum samples diluted at 1:200 were incubated with the coated plates. The optical density (OD) value for each sample was obtained at 405 nm. The cut-off value of each AAb was defined as a false positive rate of less than 5.0% based on normal controls.

### IHC

The protein expression of two TAAs (PIK3CA and SPOP) included in our panel was evaluated on a prostate tissue microarray (TMA) containing tissues from 100 PCa, 3 leiomyosarcomas, 7 simple hyperplasias, 5 cancer adjacent prostate tissues, and 6 prostate tissues, with single core per case (PR1211, TissueArray.Com). Immunohistochemistry (IHC) was performed by Boster Bio (CA, USA) following standard protocol. The PIK3CA antibody (ab135384, Abcam, USA) was diluted at 1:50, and the SPOP antibody (sc-377206, Santa Cruz Biotechnology, USA) was diluted at 1:100. The expression of two other TAAs (HSP60 and IF4H) from the panel was investigated using the Human Protein Atlas (www.proteinatlas.org), which provides accessible IHC results of protein expression profiles.

### Statistics

Data processing, hypothesis tests, and graphics were performed using Microsoft Excel, IBM SPSS Statistics 28.0, R 4.2, and GraphPad Prism 10.0. Statistical differences among groups were tested by the Mann-Whitney test, Kruskal–Wallis test, and Chi- Square test where applicable. The diagnostic value of AAbs was evaluated by the receiver operating characteristics (ROC) curve analysis. The least absolute shrinkage and selection operator (LASSO) was applied for the feature selection to select the most important AAbs. Three classification models including logistic regression (LR), random forest (RF), and support vector machine (SVM) were trained and validated on the whole data set along with 10-fold cross-validation. The best model among those three was selected for further evaluation. A two-sided *P* value with an FDR correction <0.05 was considered as a statistical difference.

## Results

### Study strategy

**Fig. 1:**
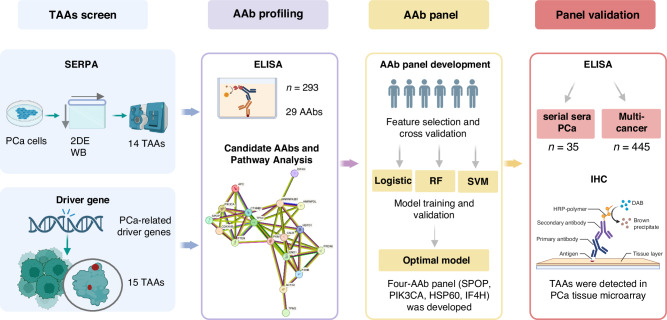
Study strategy. TAAs, tumour-associated antigens; AAbs, autoantibodies; SERPA, serological proteome analysis; 2DE, two-dimensional electrophoresis; RF, random forest; SVM, support vector machine. Created in BioRender.com.

To comprehensively identify AAb signatures in PCa, we applied a five-step strategy (Fig. 1). First, PCa-related TAAs were identified based on SERPA and cancer driver genes. TAAs showing strong immunoreactivity with PCa sera or TAAs encoded by genes that are highly mutated in PCa were identified. Second, the presence of AAbs to these identified TAAs was determined in sera of PCa and NC by indirect ELISA. The diagnostic performance of AAbs was further evaluated. Third, to enhance the performance of these AAbs in detecting PCa, an AAb panel with high accuracy was developed. Fourth, we explored the association of this panel with PCa surgery (prostatectomy) and its performance among six common cancers simultaneously. Finally, the tissue expression of selected TAAs from the panel was confirmed by IHC.

### Candidate TAAs were screened based on SERPA and cancer driver genes

**Fig. 2:**
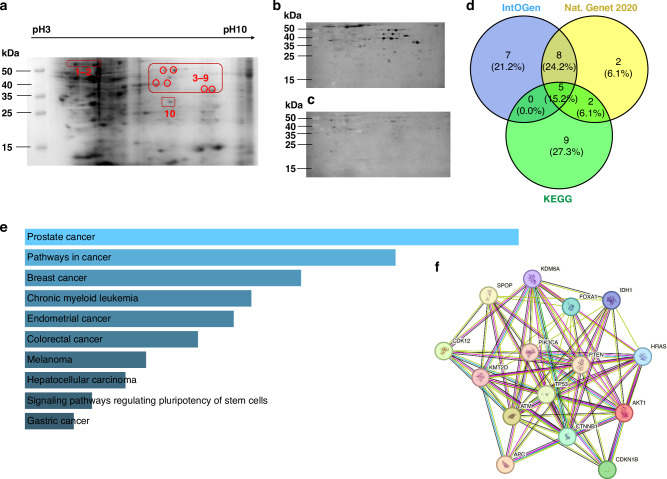
Candidate TAAs screened by SERPA and cancer driver genes strategies. **a–c** representative 2DE gel of LNCaP cellular lysates with corresponding immunoblots. A total of 30 positive spots were selected from lysates derived from the three cell lines (10 for each cell line). **a** Representative Coomassie stained 2DE gel of LnCaP cellular lysates. **b** Representative immunoblot of PCa serum pool. **c** Representative immunoblots of NC serum pool. **d**,** e** Pathway analysis of identified TAAs encoded by cancer driver genes. Three databases were used to filter PCa-related driver genes (**d**). The filtered 15 driver genes were mainly enriched in PCa pathways (**e**) and were part of the same protein-protein interaction network (**f**).

Since we hypothesised that altered proteins in PCa may serve as TAAs leading to the production of AAbs, our study initially focused on identifying overexpressed and mutated proteins in PCa. We first used the SERPA approach on three cell lines (PC3, 22Rv1, LNCaP) to identify potentially overexpressed TAAs. Whole proteins from the three cell lines were separated by 2DE, transferred to nitrocellulose, and incubated with PCa sera and NC sera. Strong autoantibody-antigen bounds (positive spots) were observed in membranes incubated with PCa sera, while membranes incubated with NC sera showed weak spots or no spots (representative SERPA results shown in Fig. 2a–c). A total of 30 positive spots were selected from the three cell lines (10 for each cell line) and analysed by MS. Fourteen immunoreactive proteins were identified, with HSP60, ENO1, and PDIA1 observed in all three PCa cell lines (Table S2). To identify mutated proteins related to PCa, cancer driver genes were analysed to find those that have high mutations and enrichment in PCa. Public databases were used to filter targeted genes, as shown in Fig. 2d, we selected 15 driver genes that were present in at least two screening strategies. The *TP53* and *SPOP* genes showed relatively high rate of mutations in PCa patients (Table S3). Functional enrichment analysis was performed to explore 15 DRPs that showed high enrichment in PCa pathways and high protein-protein interactions (PPI) with each other (Fig. 2e, f). Finally, a total of 29 candidate TAAs were identified from the two screening strategies, SERPA and analysis of cancer driver genes.

### Anti-TAAs AAbs profiling in serum samples

**Fig. 3:**
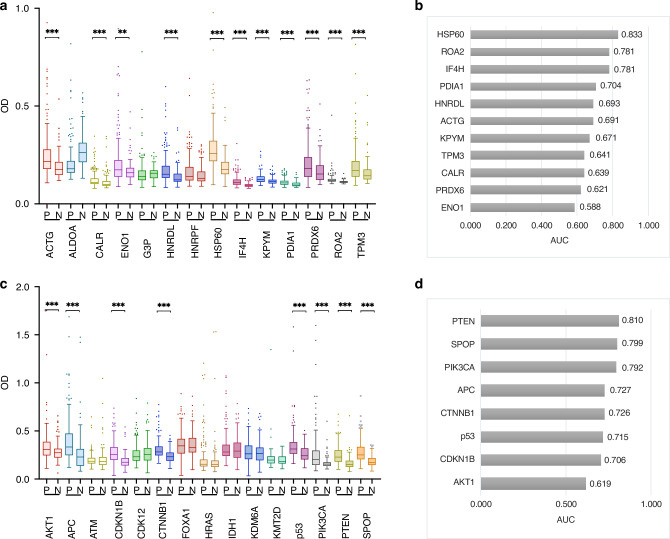
Autoantibody profiling of 29 TAAs in PCa sera. **a** Box plots of 14 AAbs identified by SERPA strategy. **b** AUCs of the 11 AAbs. **c** Box plots of 15 AAbs identified by the cancer driver genes strategy. **d** AUCs of the 8 AAbs. OD optical density, AUC area under the curve; **P < 0.01; ***P < 0.001.

**Fig. 4:**
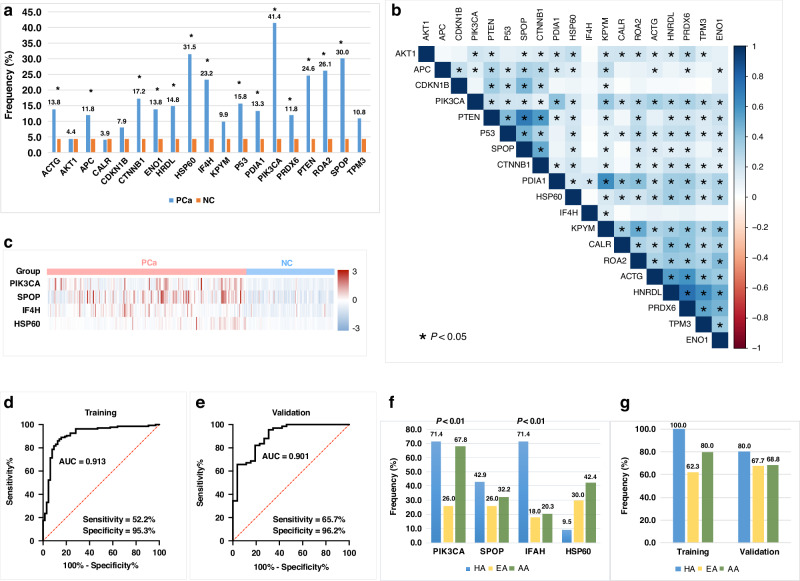
Frequencies of 19 AAbs in PCa patients and control group and development of AAb panel. **a** Frequencies of 19 AAbs in PCa and NC groups. The cut-off value was defined as a false positive rate < 5% in NC group. A significant difference was observed between PCa and NC group for 14 AAbs (* *P* < 0.05, chi-square test). **b** Correlation analysis of 19 AAbs. **c** Heatmap of four AAbs selected by the logistic regression (LR) model for developing a panel. The Z-scores of OD values from individual serum samples were used to graph a heatmap for comparability. **d**,** e** ROC analysis of the four-AAb panel in training and validation sets. **f** Frequencies of individual AAbs in the four-AAb panel in patients with PCa (Chi-square test, Bonferroni correction). **g** Frequencies of the four-AAb panel in patients with PCa. A patient was considered positive to the four-AAb panel if the patient showed positive reactivity for any of those four autoantibodies. The training group contained 11 HA, 69 EA, 40 AA patients, where 11 HA, 43 EA, and 32 AA patients showed positive reactivity for the AAb panel. There were 10 HA, 31 EA, and 16 AA patients included in the validation group, where 8 HA, 21 EA, and 11 AA patients showed positive reactivity for the AAb panel. AUC area under the curve, HA Hispanic Americans, EA European Americans, AA African Americans.

To detect the prevalence of AAbs targeting 29 candidate TAAs in PCa sera, ELISA profiling was performed on 203 PCa sera and 90 NC sera. Among the targets found in SERPA, 11 out of 14 AAbs showed significantly high levels in the PCa group compared to the NC group (*P* < 0.05, Fig. 3a). The diagnostic performance of these 11 AAbs was reflected by the area under the ROC curve (AUC). The AUCs of these AAbs ranged from 0.588 to 0.833, four of which showed an AUC > 0.700 (Fig. 3b). The HSP60 AAb showed the highest AUC of 0.833, followed by AAbs to ROA2 and IF4H, both of which showed an AUC of 0.781 in distinguishing PCa from NC. The glycolytic enzyme enolase-1 (ENO1), while having a significantly higher AAb prevalence in the PCa group compared to the NC group, had the lowest AUC (0.588) in the group. Next, we evaluated 15 target TAAs identified from cancer driver genes and found that 8 out of 15 AAbs reacting with these TAAs had higher levels in the PCa group compared to NC group (*P* < 0.05, Fig. 3c). Their AUCs ranged from 0.619 to 0.810, with AAb to PTEN showing the highest AUC (0.810), followed by AAbs to SPOP (AUC 0.799) and PIK3CA (AUC 0.792) (Fig. 3d). Taken together, these results showed that we were able to identify 19 AAbs that have significantly higher levels in PCa sera compared to NC sera. Functional enrichment analysis among the 19 TAAs targeted by these AAbs was explored by STRING and showed significant PPI enrichment (Fig. S2A). The molecular function enrichment revealed that they were involved in protein, enzyme, and RNA binding (Fig. S2B). They were also enriched in pathways associated with cancer including PCa (Fig. S2C). The 19 target TAAs were mainly involved in the biological process of regulating epithelial cell proliferation in prostate gland development (Fig. S2D). After a cut-off value was set to secure a false positive rate of < 5% in the NC group, 14 AAbs showed significantly higher frequencies in the PCa group than in the NC group (Fig. 4a). These were ACTG, APC, CTNNB1, ENO1, HNRDL, HSP60, IF4H, P53, PDIA1, PIK3CA, PRDX6, PTEN, ROA2, and SPOP. The top three most prevalent AAbs were PIK3CA, HSP60, and SPOP, which showed frequencies of 41.4%, 31.5%, and 30.0% in the PCa group, respectively.

### Evaluation of a four-AAb panel with high sensitivity in racially/ethnically diverse PCa patients

To enhance PCa detection, we developed an optimal panel with selected AAbs while maintaining relatively high sensitivity. We first explored the correlations among significant AAbs to check their co-occurrence in PCa patients (Fig. 4b). Most of them were positively correlated with each other, especially PTEN with SPOP, and HNRDL with PRDX6 (Fig. 4b). This indicates that some AAbs are co-expressed in the same serum of PCa patients. Therefore, we leveraged three classification models (RF, LR, and SVM) to select the most important AAbs that have weak correlations with each other. The data was divided into training (70%) and validation (30%) sets. The models RF, LR, and SVM selected 10, 4, and 7 AAbs, respectively with AUCs of 0.889, 0.901, and 0.885 for differentiating PCa from NC in the validation set. Finally, the LR model was used as it selected the least number of AAbs (PIK3CA, SPOP, IF4H, and HSP60) while showing the highest AUC. As depicted in Fig. 4c, all four AAbs showed high Z-scored OD values in the PCa group. Additionally, the LR model was stable in both the training and validation sets, showing an AUC of 0.913, with 52.2% sensitivity and 95.3% specificity in the training set, and 65.7% sensitivity and 96.2% specificity in the validation set (Fig. 4d, e).

Next, we evaluated the four-AAb panel in PCa patients from different backgrounds, including self-identified 21 Hispanic Americans (HA), 100 European Americans (EA), and 59 African Americans (AA). The frequencies of individual AAbs in these three populations are shown in Fig. 4f. AAb to PIK3CA exhibited significantly higher frequencies in HA and AA patients compared to EA patients. The IF4H AAb also showed significantly higher frequency in HA patients compared to EA and AA, while AAbs to SPOP and HSP60 showed no significant difference among groups (Fig. 4f). However, no significant differences were observed among the three patient groups when the four AAbs were combined as a panel (Fig. 4g).

### Performance of the AAb panel in serial and multi-cancer sera

**Fig. 5:**
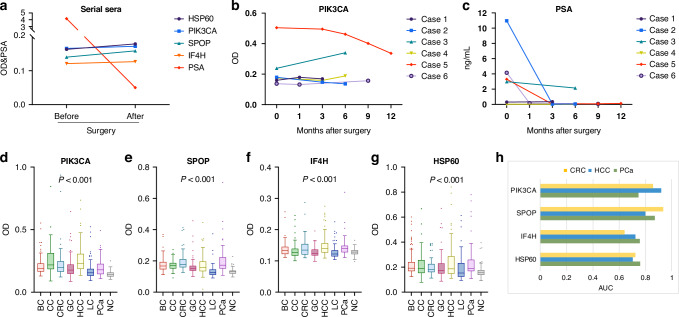
Performance of the four-AAb panel in serial and multi-cancer sera. **a** Trends of levels of four AAbs and PSA before and after surgery in PCa patients. OD values of AAbs and PSA levels (ng/mL) were indicated on the Y-axis. Median OD values and median PSA levels were used to depict the trends before and after surgery. **b**,** c** Levels of PIK3CA and PSA after surgery in representative PCa patients over time. **d–g** Box plots of the four AAbs in multiple cancers. **h** AUCs of the four AAbs in the top three cancers. OD optical density, PSA prostate-specific antigen, AUC area under the curve, CRC colorectal cancer, HCC hepatocellular carcinoma.

To explore the association of the AAb panel with prostate surgery, the four AAbs were tested in 35 serial sera from 13 PCa patients before and after surgery. PSA values for these patients were available, with 7 out of 13 (53.8%) patients showing abnormal PSA values (>4 ng/mL) before surgery, while 5 out of 13 (38.5%) patients showed positive to our panel. After combining the panel with PSA as a parallel test, it could identify 10 of 13 PCa patients (76.9%). These ten patients showed positive to either the panel or PSA. As shown in Fig. 5a, the four AAbs stayed at about the same level after surgery, while the PSA levels decreased after surgery with the median level of 4.15 ng/mL reduced to 0.05 ng/mL. To determine whether the change in individual AAb levels is variable among different patients, we randomly selected six patients to examine the trend of PIK3CA AAb levels over time after surgery. Our analysis showed that PIK3CA AAb levels slightly decreased from six months to 12 months in Case 5, however, they slightly increased in Case 3 six months after surgery (Fig. 5b). All these patients had decreased levels of PSA after surgery (Fig. 5c). We also conducted correlation analysis between PSA and each of four AAbs in the panel, three of them (PIK3CA, IF4H, HSP60) showed no correlations with PSA (Fig. S3). Only SPOP showed significant correlation with PSA (R = 0.62, *P* < 0.05). This further demonstrated its potential as a complementary biomarker to PSA for enhancing PCa detection.

Since the identified AAbs are highly related to cancers [30–32], we questioned whether they have the potential for multi-cancer detection. Therefore, 445 serum samples from six common cancers (BC, CC, CRC, GC, HCC, LC), as well as 70 randomly selected PCa were used to test the four-AAb panel simultaneously. As shown in Fig. 5d–g, except for LC, the other cancers showed significantly high AAb levels compared to NC group (non-parametric test and post-hoc test, *P* < 0.05, adjusted by Bonferroni correction). The top three cancers are HCC, PCa, and CRC due to their high median OD values for the four AAbs. The diagnostic performance of the four AAb panel in the top three cancers was evaluated by ROC analysis, AAbs to IF4H and HSP60 had the highest AUCs of 0.756 and 0.759 in PCa, respectively (Fig. 5h). The AAb to SPOP showed the best performance in CRC (AUC = 0.935) followed by PCa (AUC = 0.871), and PIK3CA AAb had an AUC of 0.920 in HCC, followed by CRC (AUC = 0.857) and PCa (AUC = 0.747).

### TAAs expression in PCa tissues

**Fig. 6:**
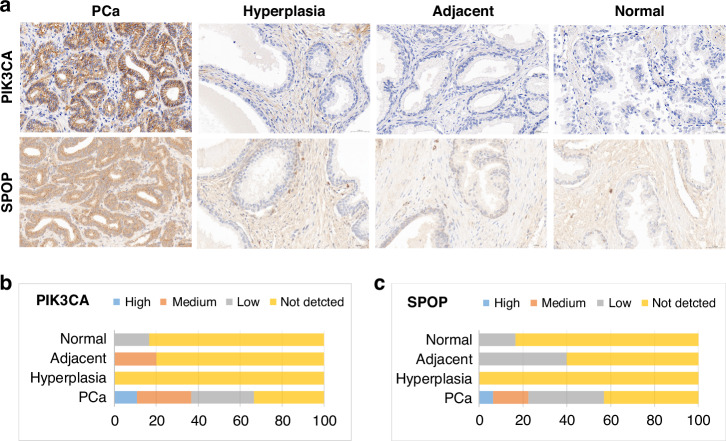
TAA protein expressions in PCa tissues. **a** Representative IHC staining of PIK3CA and SPOP in PCa (40 x magnification), benign prostatic hyperplasia, adjacent to the tumour, and normal prostate tissues. **b**,** c** percentage of high to low stained tissues in different tissue types.

Two of the four AAbs in our final panel were derived from the SERPA strategy (HSP60, IF4H), which identified these TAAs at the protein level. However, since the other two AAbs, PIK3CA and SPOP, were screened via the cancer driver gene strategy at the gene level, we performed IHC to examine their antigen expression in PCa tissues (100 PCa tissues total, 93 available) and control tissues. We observed high staining of both two TAAs in PCa tissues, whereas they were either not detected or exhibited low staining in prostate hyperplasia, adjacent, and normal tissues. (Fig. 6a–c). PIK3CA showed high to low cytoplasmic and membranous staining in more than 60.0% of PCa tissues. There were 10.8% (10/93), 25.8% (24/93), and 30.1% (28/93) of PCa tissues that showed high, medium, and low staining to PIK3CA, respectively. Only 1 of 5 adjacent tissues and 1 of 6 normal tissues showed medium or low staining, respectively. SPOP showed more than 50.0% high to low cytoplasmic staining in PCa tissues. The background staining of SPOP was higher than that of PIK3CA. SPOP showed 6.5% (6/93), 16.1% (15/93), and 34.4% (32/93) of high, medium, and low PCa tissue staining, respectively. The adjacent and normal tissues showed 2 out of 5 and 1 out of 6 with low SPOP staining, respectively. Neither PIK3CA nor SPOP were detected in 7 prostatic hyperplasia tissues (Fig. 6b, c). The three leiomyosarcoma tissues showed negative staining of PIK3CA, as well as one medium and one low SPOP staining. Additionally, we explored the tissue expression of HSP60 and IF4H in the HPA database (www.proteinatlas.org). There were 9 out of 10, and 1 out of 10 PCa tissues showing high and medium staining of HSP60, respectively. IF4H showed medium staining in 10 out of 12 PCa tissues.

## Discussion

This study identified TAAs and corresponding AAbs in PCa and evaluated the diagnostic potential of the AAbs. We performed a comprehensive screening of TAAs using SERPA and cancer driver genes strategies and identified overexpressed proteins and DRPs that held high immunogenicity resulting in high levels of AAbs in serum samples of PCa. An AAb panel containing four AAbs (PIK3CA, SPOP, IF4H, HSP60) was developed, which significantly improved the detection of PCa compared to individual AAbs. This panel could complement existing biomarkers such as PSA to facilitate non-invasive PCa detection with high sensitivity. We also observed that individual AAb responses to the four selected TAAs varied by race/ethnicity, with PIK3CA significantly targeted by AAbs at higher frequencies in both HA and AA patients and IF4H preferentially targeted in AA patients. Although not statistically significant, we detected a trend towards higher frequency of AAbs to SPOP in HA and AA patients, and of AAbs to HSP60 in AA patients. Additionally, we found that AAbs from the panel were also significantly expressed in five common cancers (BC, CC, CRC, GC, HCC), suggesting that this panel has the potential for multi-cancer detection. The expression of the proteins in the panel were also confirmed in PCa tissues, indicating the consistency of high autoantibody-antigen expression.

The SERPA approach has been used as an invaluable tool to identify biomarkers among various diseases [33, 34]. It is a robust technique for immunoreactive antibody-antigens identification with high sensitivity. By performing this approach, we were able to identify 11 AAb-TAAs in PCa. These AAbs were significantly highly expressed in PCa sera compared to controls, especially the anti-HSP60 AAb response was identified in all three PCa cell lines and showed the best classification performance with an AUC of 0.833, a sensitivity of 31.5%, and 95.6% specificity. This finding was supported by our previous study, which reported a good diagnostic performance for HSP60 AAb (AUC = 0.863) in a Luminex AAb panel [23]. Moreover, the HSP60 protein is highly expressed in PCa tissues and plays an essential role in prostate tumorigenesis [35, 36]. Its overexpression has been shown to be positively correlated with high Gleason score in AA patients with PCa [37], which would be consistent with the observed trend towards high frequency of the anti-HSP60 AAb in our AA patient group. In addition, an AAb response against ENO1 was also detected in all three cell lines, consistent with a previous study highlighting the strong association between ENO1 AAb and PCa [38]. Other studies reported autoantibodies against AMACR and HIP1 by using protein microarray and mice model methods [39]. They showed potential in distinguishing PC patients from healthy controls, with AMACR exhibited 71.8% sensitivity, 61.6% specificity, while HIP1showed 88% sensitivity and 64% specificity[39]. Although the two markers achieved high sensitivity, the low specificity may cause an increased false positive rate (FPR = 100-Specificity), potentially lead to overdiagnosis and overtreatment. In our study, we controlled a specificity greater than 95% (FPR < 5%). Our results support the hypothesis that abnormally expressed proteins could trigger immunoreaction leading to AAbs production.

A limitation of the SERPA approach is the reliance on 2DE gel electrophoresis. The ability of this system to separate post-translationally modified protein isoforms or some cell membrane proteins remains challenging [33]. In our study, we used another strategy – analysis of cancer driver genes encoding TAAs- to further improve the possibility of identifying potential TAAs that SERPA may not discern. Driver genes are typically mutated in cancer patients and drive tumorigenesis [20]. Proteins encoded by these genes may be closely related to cancer and trigger the immune response of autoantibodies. A wide screening of cancer driver genes with high mutations and enrichment in PCa was performed based on three databases [20, 21, 27]. The top 15 driver genes that were highly related to PCa were selected for further analysis of AAb responses. We used ELISA to test the prevalence of serum AAbs against 15 DRPs in PCa. Eight AAbs showed significantly high levels in the PCa group compared to controls. Among these, the AAb to PIK3CA showed the highest frequency of 41.4%, followed by the SPOP AAb (30.0%). Notably, the *SPOP* gene is a tumour suppressor that has a high mutation rate of 8.9% in PCa patients (Table S3). *PIK3CA* mutations have also been correlated with poor PCa progression, and the immunogenicity of antigens from mutated PIK3CA has been reported [40, 41]. Our findings suggested that mutated proteins, especially DRPs (driver gene-related proteins), could become TAAs with high immunogenicity triggering circulating AAbs. However, the mutation information was obtained from public databases, we have not tested the specific mutation of those driver genes in our PCa samples. Future studies may focus on analysing certain mutations and AAb levels in the same patient.

Previous findings indicated that certain AAbs were more prominent in PCa patients from specific racial or ethnic backgrounds, suggesting differences in immunobiology between PCa populations from different backgrounds [23, 38, 42, 43] In this study, the frequency of anti-PIK3CA AAbs was significantly elevated in HA and AA PCa patients compared to EA patients. It is not clear if this elevated frequency correlates with *PIK3CA* mutation rates in HA and AA patients since these rates have not been reported in HA patients and have been found to be very low in some AA PCa patient cohorts [44, 45]. While the anti-IF4H AAb frequency was significantly elevated in HA patients, its biological and health disparity significance are unclear due to the scarcity of studies on the mutation rates of the *EIF4H* gene in PCa patients. The SPOP AAb showed slightly higher frequency in AA and HA patients. This might be related to the higher prevalence (up to ∼30%) of *SPOP* gene in AA patients [46, 47]. The high mutation rate could generate an altered SPOP protein capable of triggering an autoantibody response in different racial/ethnic groups as observed in our study. Whether the elevated trend in the frequency of anti-SPOP AAbs observed in HA and AA patients would reach significance in larger cohorts and correlate with the SPOP mutation frequency remains to be investigated. Taken together, the observed differences among the HA, AA, and EA groups in AAb frequencies could be related, in addition to mutation rates, to other differences in genetic and molecular pathways reported in these three distinct PCa populations [48]. However, our findings need to be validated in future studies with larger cohorts because the sample size of our HA group was relatively small (21 samples), likely causing sampling bias.

The identified 19 AAb-targeting TAAs are closely associated with PCa. Functional analysis revealed that all 19 proteins showed high enrichment in PPI networks and in pathways associated with cancer including PCa, proliferation, and regulation of epithelial cells involved in prostate gland development, functioning as protein binding, and enzyme binding. Across the identified 19 AAbs, some positively correlated with each other, meaning that they co-expressed in the same patient serum sample. The sensitivity of individual AAb for distinguishing PCa from non-PCa is usually low and may not meet the criteria for clinical application. However, multi-AAb panels can enhance cancer detection by improving diagnostic sensitivity and specificity [26, 49, 50]. In this study, we leveraged three classification models (LR, RF, SVM) to develop a cost-effective multi-AAb panel using fewer AAbs to achieve high sensitivity. An optimal AAb-panel that contains four AAbs (PIK3CA, SPOP, IF4H, HSP60) was finally developed by using LR model. The LR model demonstrated the best trade-off between predictive performance and model simplicity, achieving the highest AUC (0.901) while selecting only four AAbs (Table S4). This model not only simplifies clinical translation but also reduces the risk of overfitting, especially in studies with limited sample sizes. Furthermore, the selected AAbs exhibited low correlation with each other, indicating that LR effectively selected non-redundant biomarkers that provide complementary diagnostic information. Compared to RF and SVM, which identified larger panels with similar or lower AUCs, LR’s performance supports its robustness and relevance for potential clinical application. The final panel showed high classification performance with AUCs of 0.913, and 0.901 in training and validation sets, respectively. It had a sensitivity of 65.7% in distinguishing PCa from NC with only a 3.8% false positive rate (96.2% specificity).

A previous study by our group reported an autoantibody panel consisting of seven targets (cyclin B1, Survivin, p53, RalA, DFS70/LEDGFp75, MDM2, and NPM1) that could discriminate between PC patients and healthy controls with 80.5% sensitivity and 91% specificity [51]. In our present study, the AAb panel could achieve a higher specificity by using only four AAbs. Although our previous study reported higher sensitivity, rather than merely combining seven AAbs, we used in the present study various classification models to identify the most significant AAbs, which could enhance diagnostic efficiency while reducing the cost of testing unnecessary biomarkers. We also examined the correlation between PSA levels and each of the four autoantibodies in the final panel (Fig. S3). Autoantibodies to PIK3CA, IF4H, HSP60 showed no significant correlation with PSA, suggesting that these markers were not co-expressed in the same PCa patients and may offer independent, complementary diagnostic value. A significant correlation was observed between SPOP and PSA, which might reflect the strong biological association of SPOP with PCa. The *SPOP* gene is the most frequently mutated gene in PCa, and our results showed that 30.0% of PCa cases exhibited positive expression of SPOP autoantibody with 96.4% specificity. While promising, this finding warrants further validation in larger cohorts due to the limited sample size in the current study.

Blood-based multi-cancer detection (MCD) has been developed to detect multiple cancers in a single test [52, 53]. This allows for rapid and low-cost screening of patients with a high risk of cancer [53]. To explore the MCD potential of the four-AAb panel, we tested the panel in seven cancers simultaneously. The levels of all four AAbs in the panel were significantly elevated in 6 out of 7 cancers. The panel showed medium to high classification performance in HCC, CRC, and PCa. The AAbs to HSP60 and IF4H, which were identified by the SERPA strategy, showed the highest AUC in distinguishing PCa from NC. They were more specific to PCa because they were identified by probing whole cell lysates from PCa cell lines with AAbs in immunoblots. Although the other two AAbs (PIK3CA, SPOP), identified by the cancer-driver gene strategy, showed the best performance in either HCC or CRC, they also had high AUCs in PCa. *PIK3CA* is a common cancer driver gene, and its mutation has been reported in HCC, where this gene plays an important role in malignant development [54, 55]. Also, reports show that *SPOP* mutations are associated with CRC [56, 57]. These cancer driver genes are involved in the development of multiple types of cancer. These findings suggested that a high abundance of certain circulating AAbs may be associated with the high mutations in driver genes encoding their target TAAs in specific cancers. Taken together, our results also suggest that the four-AAb panel has the potential for multi-cancer detection.

To explore if the four target TAAs, which induced high levels of AAbs, were also highly expressed in PCa tissues, we used IHC analysis on prostate tissue arrays and publicly available IHC datasets (HPA database). The IHC results showed strong staining for PIK3CA and SPOP in PCa tissues. However, the background for SPOP staining was high (low signal-to-noise ratio), which might be caused by the low specificity of the SPOP antibody. A previous IHC study focused on SPOP expression in tissues corresponding to primary PCa, castration-resistant prostate cancer (CRPC), benign prostatic hyperplasia, and normal prostate, showed that SPOP expression remained unchanged in tumour tissues relative to benign tissues [58]. The PIK3CA protein expression was reported as significantly higher in PCa tissues than in non-cancer tissues [59]. We also found medium to strong IHC staining of IF4H and HSP60 in the HPA database. Therefore, the observed high levels of circulating AAbs to these TAAs were consistent with their elevated tissue expression in PCa.

In summary, the results from this study strongly suggested that altered proteins that are highly expressed in PCa tissues may trigger high serum levels of AAbs, and these AAbs could serve as blood-based biomarkers to facilitate PCa detection. In addition, differences in AAb responses among distinct ethnic population may reflect differences in the biology of their target TAAs (e.g., mutational rate, expression, functionality, protein-protein interactions).

Although our study was comprehensive in identifying AAbs to altered proteins in PCa, it still has some limitations, the main one being the limited sample size which may not be sufficient to allow accurate clinical interpretations supported by very high sensitivity and specificity. However, since this is a retrospective study, the clinical usage of our four-AAb panel can be validated in future prospective cohort studies. The identified AAbs were not specific to PCa, limiting their potential application as independent biomarkers. They can be complementary tools to combine with specific markers such as PSA, AFP, or CEA. In addition, the SERPA strategy used in this study also has drawbacks. First, 2-DE usually can only find relatively abundant proteins, and it’s limited to separate proteins that co-migrate due to post-translational modifications. Second, even with advances in 2-DE-compatible detergents, the separation of membrane proteins remains challenging due to their poor solubility in aqueous buffers. Lastly, challenges in 2-D gel reproducibility and time-consuming limit its widespread application [60].

In conclusion, this study identified AAbs-TAAs biomarkers and developed a four-AAb panel to enhance the accuracy of PCa immunodiagnosis. The panel also has the potential for multi-cancer detection. The strategies used in this study and the identified AAbs-TAA systems could be applied in future studies to investigate novel targets for PCa prevention or treatment.

## Supplementary information

Supplementary Materials

Original gel and immunoblotting

## Data Availability

Data is available upon request from the corresponding author.
