# Supplementary material for: A panel of four autoantibodies to tumour-associated antigens in patients with prostate cancer and its potential for multi-cancer detection
Source: Br J Cancer. 2025 Nov 18;134(3):493–503. doi: 10.1038/s41416-025-03242-8 (PMC12852787; doi:10.1038/s41416-025-03242-8)
Supplement: Supplementary file 1 — Supplementary Materials [file 41416_2025_3242_MOESM1_ESM.docx]

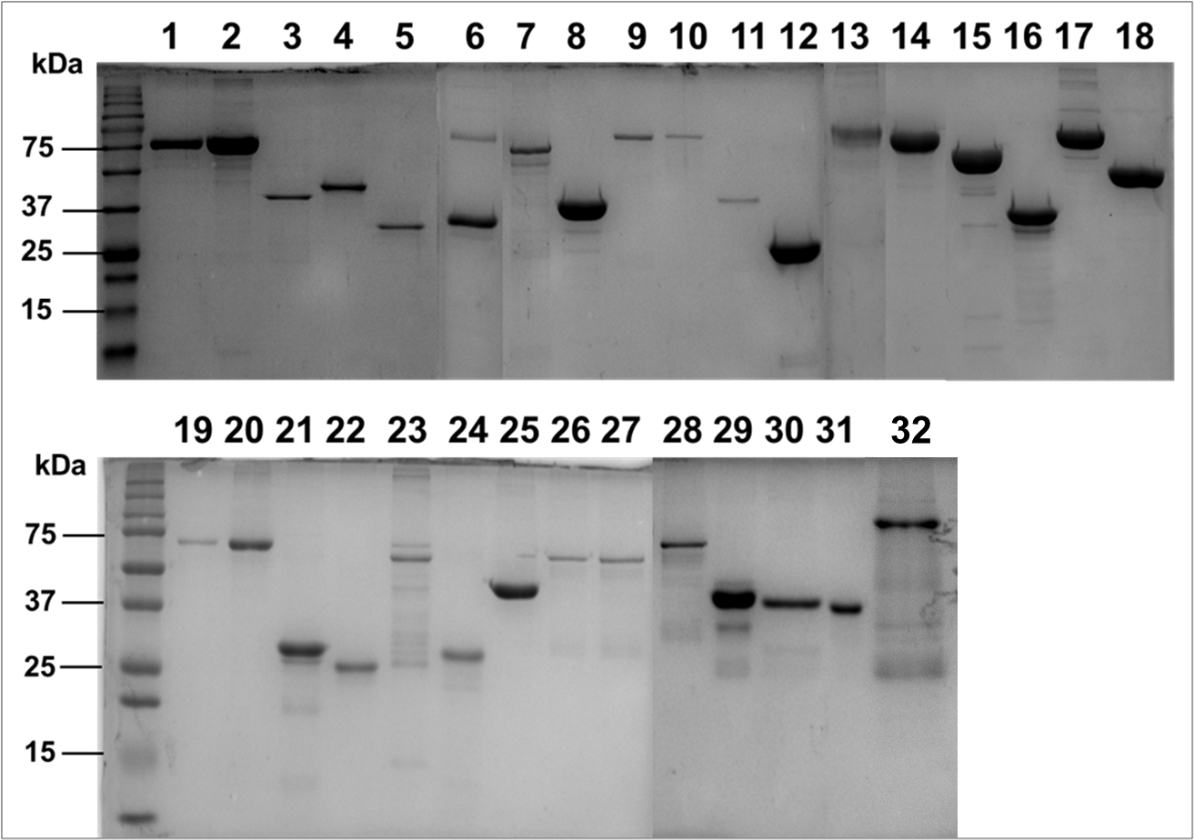


**Figure S1**. SDS-PAGE gel confirmation of purifications of purchased proteins. Lanes 1, 2, 19, and 20 were loaded with BSA as loading controls. 3, PIK3CA; 4, SPOP; 5, TPM3-V1; 6, TPM3-V2; 7, ROA2; 8, G3P; 9, HNRPF; 10, ACTG1; 11, HNRDL; 12, PRDX6; 13, CALR; 14, P4HB; 15, ENO1; 16, IF4H; 17, KPYM; 18, ALDOA; 21, APC; 22, HRAS; 23, ATM; 24, CDKN1B; 25, KDM6A; 26, AKT1-V1; 27, AKT1-V2; 28, IDH1; 29, CDK12; 30, KMT2D; 31, FOXA1; 32, CTNNB1.


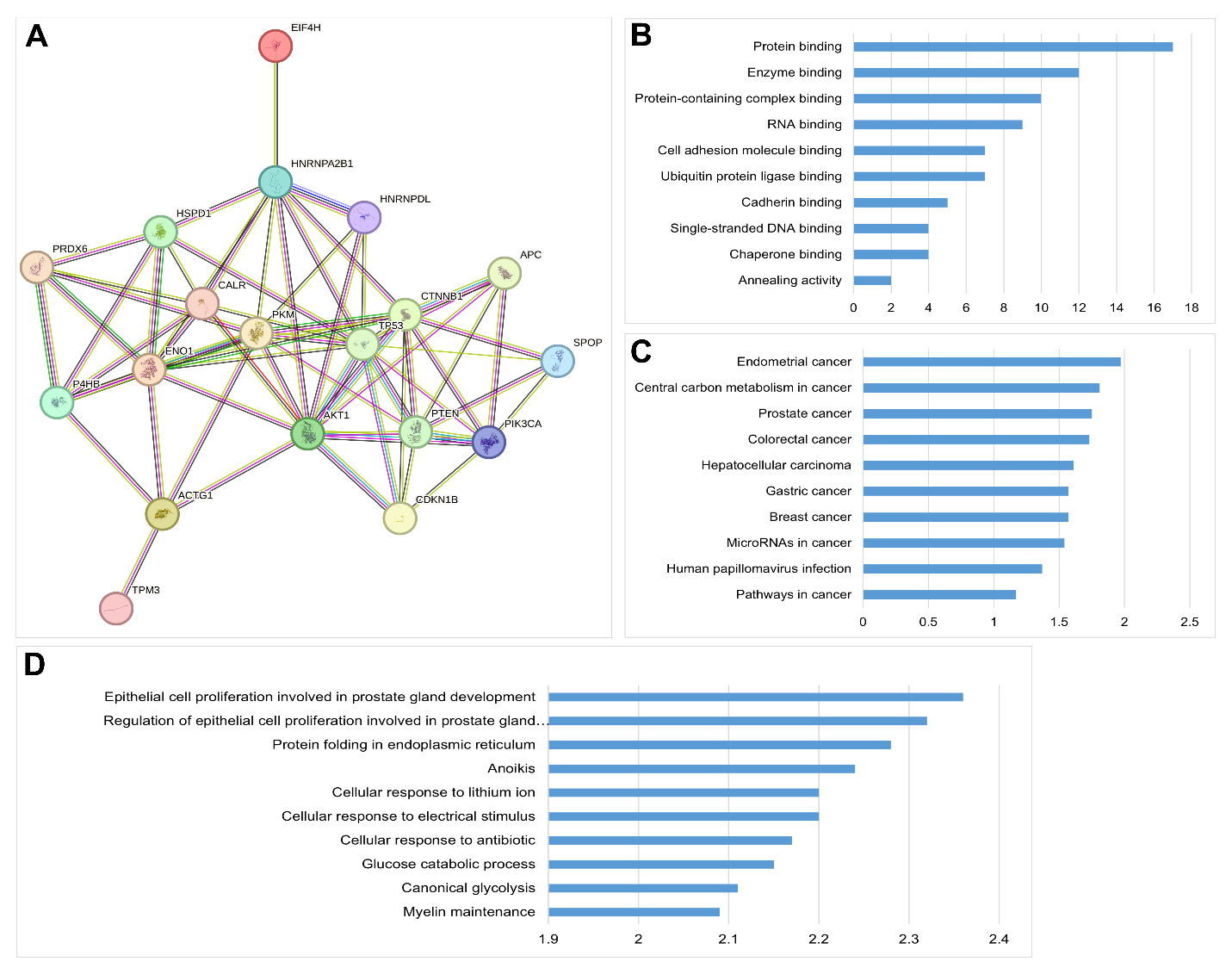
**Figure S2**. PPI analysis of the identified 19 TAAs. A, protein-protein interaction (PPI) of TAAs. B, molecular function of TAAs. C, KEGG pathway of TAAs. D, Biological process of TAAs.


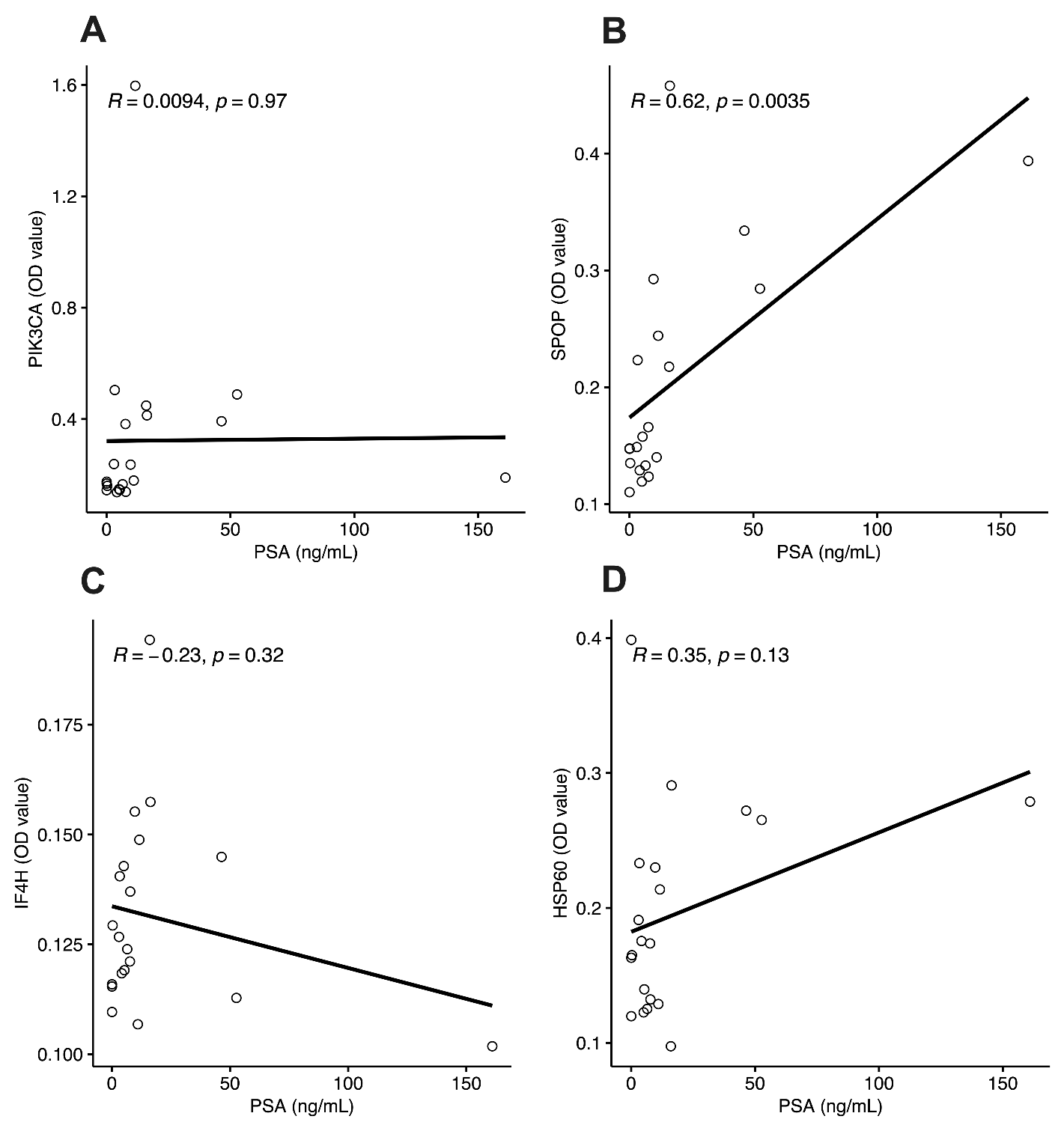


**Figure S3**. Correlation analysis of PSA and four autoantibodies from the final panel (Pearson correlation analysis). Patients with both PSA level and autoantibody values available were used to explore the commentary potential of the identified autoantibody panel.

**Table S1**. Information of recombinant proteins used for ELISA test.

| **Method** | **Protein** | **Provider** | **Length** | **Amino Acids Sequence** | **Expression System** | **Purification (%)** |
| --- | --- | --- | --- | --- | --- | --- |
| **SERPA** | ACTG1 | Novus biological | Full | 1-375 | Wheat germ | > 80% |
|  | ALDOA | Novus biological | Full | 1-365 | *E.coli* | > 95% |
|  | CALR | Abcam | Full | 18-417 | *E.coli* | > 90% |
|  | ENO1 | Novus biological | Full | 1-434 | *E.coli* | > 95% |
|  | G3P | Novus biological | Full | 1-335 | *E.coli* | >95% |
|  | HNRDL | Novus biological | Partial | 301-409 | *E.coli* | > 90% |
|  | HNRPF | Novus biological | Full | 1-415 | Wheat germ | > 80% |
|  | HSP60 | Abcam | Full | 1-573 | *E.coli* | > 90% |
|  | IF4H | Novus biological | Full | 1-248 | *E.coli* | > 90% |
|  | KPYM | Novus biological | Full | 1-531 | *E.coli* | > 95% |
|  | PDIA1/P4HB | Abcam | Full | 1-508 | *E.coli* | > 90% |
|  | PRDX6 | Novus biological | Full | 1-224 | *E.coli* | > 95% |
|  | ROA2 | Abcam | Full | 1-249 | *E.coli* | > 90% |
|  | TPM3 | Novus biological | Partial | 1-248 | *E.coli* | > 85% |
| **DRPs** | AKT1 | Abcam | Full | 1-480 | *E.coli* | > 95% |
|  | APC | LSBio | Partial | 2634-2843 | *E.coli* | > 95% |
|  | ATM | LSBio | Partial | 2484-2748 | *E.coli* | > 95% |
|  | CDK12 | Novus biological | Partial | 1281-1380 | Wheat germ | > 80% |
|  | CDKN1B | Ray Biotech | Full | 2-198 | *E.coli* | > 85 % |
|  | CTNNB1 | Sino Biological | Full | 1-781 | Baculovirus-Insect Cells | > 85 % |
|  | FOXA1 | Novus biological | Full | 1-472 | Wheat germ | > 80% |
|  | HRAS | LSBio | Full | 2-189 | *E.coli* | > 95% |
|  | IDH1 | Novus biological | Full | 1-414 | *E.coli* | > 90% |
|  | KDM6A | Novus biological | Partial | 641-740 | Wheat germ | > 80% |
|  | KMT2D | Novus biological | Partial | 1487-1586 | Wheat germ | > 80% |
|  | PIK3CA | COSMO BIO USA | Partial | 959-1068 | Wheat Germ | > 80% |
|  | PTEN | Cancer Autoimmunity Research Laboratory of UTEP | Full | 1-403 | *E.coli* | > 80% |
|  | P53 | Cancer Autoimmunity Research Laboratory of UTEP | Full | 1-393 | *E.coli* | > 80% |
|  | SPOP | Novus biological | Full | 1-374 | *E.coli* | > 90% |

SERPA, serological proteome analysis; DRPs, driver gene-related proteins.

**Table S2**. Proteins identified by SERPA strategy.

| Accession | UniProt ID | Full name | Protein MW (kDa) | Protein PI | Protein Score C.I.% | Cell lysates | N. Spots^*^ |
| --- | --- | --- | --- | --- | --- | --- | --- |
| ACTG_HUMAN | P63261 | Actin, cytoplasmic 2 | 42 | 5.31 | 100 | 22Rv1 | 2 |
| ALDOA_HUMAN | P04075 | Fructose-bisphosphate aldolase A | 39 | 8.3 | 100 | LNCap | 1 |
| CALR_HUMAN | P27797 | Calreticulin | 48 | 4.29 | 100 | 22Rv1 | 1 |
| CH60_HUMAN | P10809 | 60 kDa heat shock protein | 61 | 5.7 | 100 | LNCap, 22Rv1, PC-3 | 3 |
| ENOA_HUMAN | P06733 | Alpha-enolase | 47 | 7.01 | 100 | LNCap, 22Rv1, PC-3 | 10 |
| G3P_HUMAN | P04406 | Glyceraldehyde-3-phosphate dehydrogenase | 36 | 8.57 | 100 | 22Rv1, PC-3 | 2 |
| HNRDL_HUMAN | O14979 | Heterogeneous nuclear ribonucleoprotein D-like | 46 | 9.59 | 100 | PC-3 | 1 |
| HNRPF_HUMAN | P52597 | Heterogeneous nuclear ribonucleoprotein F | 46 | 5.38 | 100 | 22Rv1 | 1 |
| IF4H_HUMAN | Q15056 | Eukaryotic translation initiation factor 4H | 27 | 6.67 | 100 | LNCap | 1 |
| KPYM_HUMAN | P14618 | Pyruvate kinase PKM | 58 | 7.96 | 100 | LNCap | 2 |
| PDIA1_HUMAN | P07237 | Protein disulfide-isomerase | 57 | 4.76 | 100 | LNCap, 22Rv1, PC-3 | 3 |
| PRDX6_HUMAN | P30041 | Peroxiredoxin-6 | 25 | 6 | 100 | PC-3 | 1 |
| ROA2_HUMAN | P22626 | Heterogeneous nuclear ribonucleoproteins A2/B1 | 37 | 8.97 | 100 | 22Rv1 | 1 |
| TPM3_HUMAN | P06753 | Tropomyosin alpha-3 chain | 33 | 4.68 | 100 | PC-3 | 1 |

*, positive spots taken from gels were identified as the same protein. PI, isoelectric point.

**Table S3**. Identified PCa-related cancer driver genes.

| **Symbol** | **Gene Name** | **Gene Type** | **Core pathway** | **Process** | **Cohorts^*^** | **Samples** | **Mutated samples** | **Mutated rate (%)** |
| --- | --- | --- | --- | --- | --- | --- | --- | --- |
| *AKT1* | v-akt murine thymoma viral oncogene homolog 1 | Oncogene | AKT/PI3K | Cell Survival | 1 | 492 | 3 | 0.61 |
| *APC* | adenomatous polyposis coli | TSG | PI3K | Cell Survival | 4 | 1009 | 27 | 2.7 |
| *ATM* | ataxia telangiectasia mutated | TSG | PI3K | Cell Survival | 6 | 1255 | 35 | 2.8 |
| *CDK12* | cyclin dependent kinase 12 | Oncogene | APC | Cell Fate | 5 | 987 | 23 | 2.3 |
| *CDKN1B* | cyclin dependent Kinase inhibitor 1B | TSG | RAS | Cell Survival | 3 | 873 | 10 | 1.1 |
| *CTNNB1* | catenin beta 1 | Oncogene | WNT | Cell Fate | 3 | 756 | 14 | 1.9 |
| *FOXA1* | hepatocyte nuclear factor 3-alpha | Oncogene | PI3K | Cell Survival | 8 | 1438 | 80 | 5.6 |
| *HRAS* | v-Ha-ras Harvey rat sarcoma viral oncogene homolog | Oncogene | RAS | Cell Survival | 1 | 492 | 4 | 0.81 |
| *IDH1* | isocitrate dehydrogenase 1 (NADP+) | Oncogene | APC | Cell Fate | 2 | 604 | 7 | 1.2 |
| *KDM6A* | lysine (K)-specific demethylase 6A | TSG | TGF-beta | Cell Fate | 6 | 1235 | 21 | 1.7 |
| *KMT2D* | histone-lysine N-methyltransferase 2D | TSG | WNT | Cell Cycle | 5 | 1074 | 46 | 4.3 |
| *PIK3CA* | phosphoinositide-3-kinase, catalytic, alpha polypeptide | Oncogene | PI3K | Cell Fate | 4 | 918 | 20 | 2.3 |
| *PTEN* | phosphatase and tensin homolog | TSG | PI3K | Cell Fate | 8 | 1458 | 44 | 3 |
| *SPOP* | speckle-type POZ protein | TSG | PI3K | Cell Survival | 8 | 1343 | 119 | 8.9 |
| *TP53* | tumor protein p53 | TSG | RAS | Cell Survival | 9 | 1503 | 141 | 9.4 |

*, the number of cohorts where the targeted gene was reported (a total of nine cohorts were analyzed); TSG, tumor suppressor genes.

**Table S4**. Performance of three classification models

| **Models** | **AAbs in the model** | **AUC** |
| --- | --- | --- |
| Logistic Regression | PIK3CA + SPOP + HSP60 + IF4H | 0.901 |
| Random Forest | HSP60 + PTEN + IF4H + PIK3CA + SPOP + ROA2 + APC + CDKN1B + HNRDL + CTNNB1 | 0.889 |
| Support Vector Machine | HSP60+PTEN+SPOP+PIK3CA+IF4H+ROA2+APC | 0.885 |

AUC, area under the curve.
