# Supplementary material for: A panel of four autoantibodies to tumour-associated antigens in patients with prostate cancer and its potential for multi-cancer detection
Source: Br J Cancer. 2025 Nov 18;134(3):493–503. doi: 10.1038/s41416-025-03242-8 (PMC12852787; doi:10.1038/s41416-025-03242-8)

LNCap

Immunoblotting of sera

PCa sera

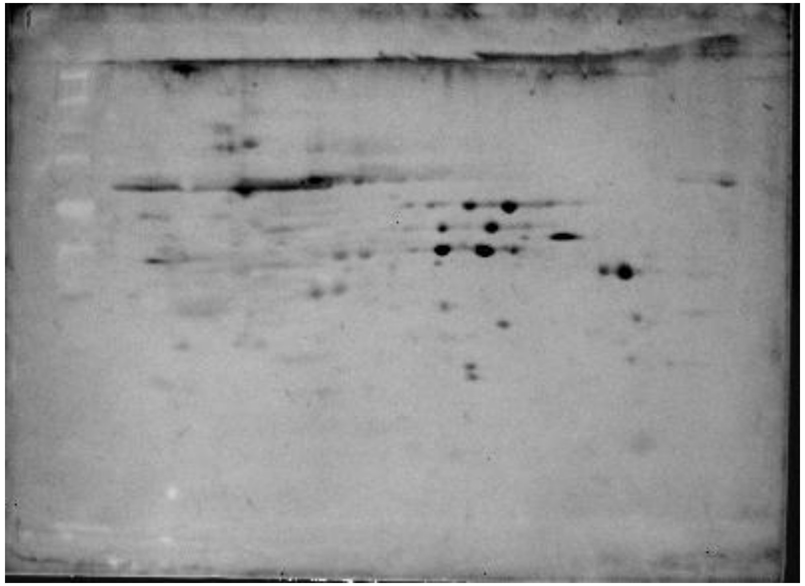

NC sera

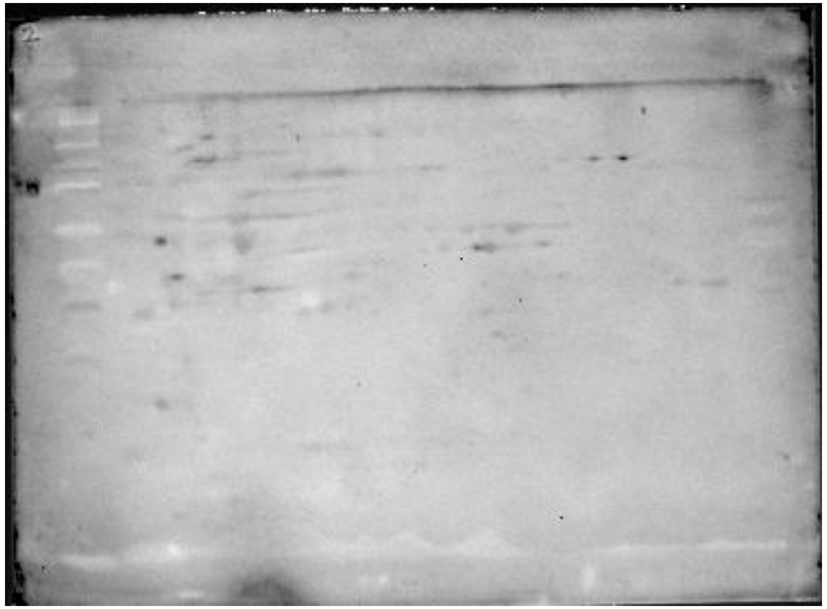

2D- Gel of LNCap

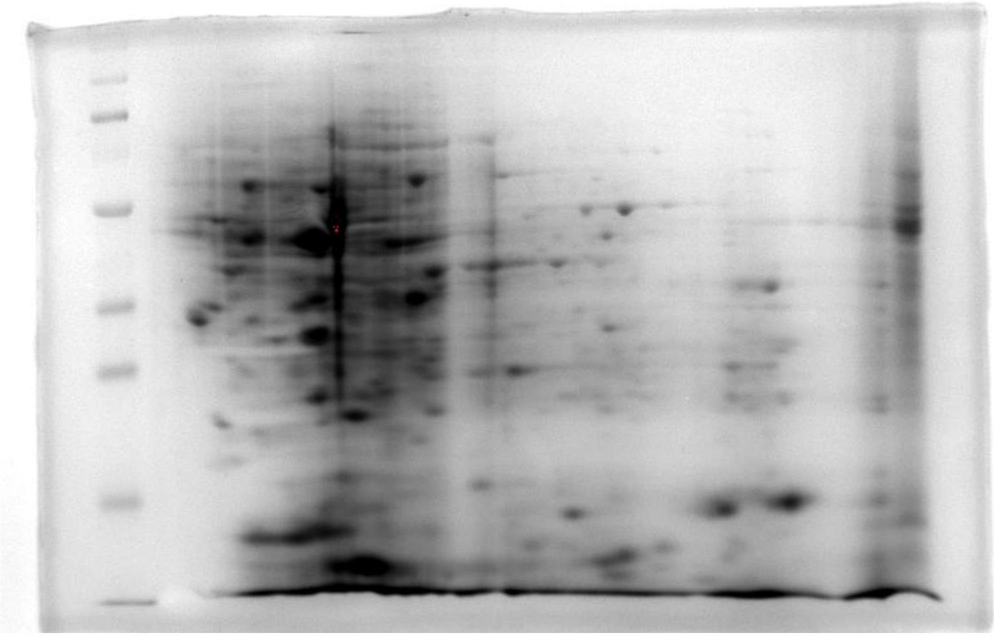

22Rv1

Immunoblotting of sera

PCa sera

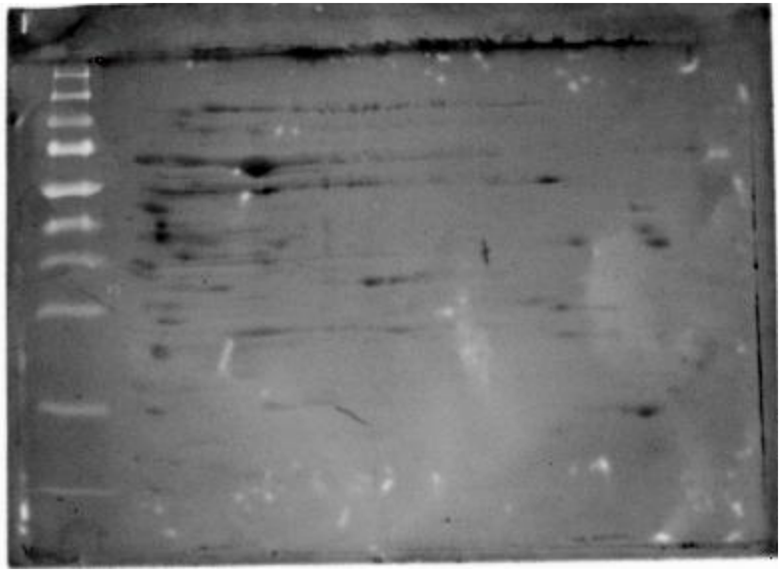

NC sera

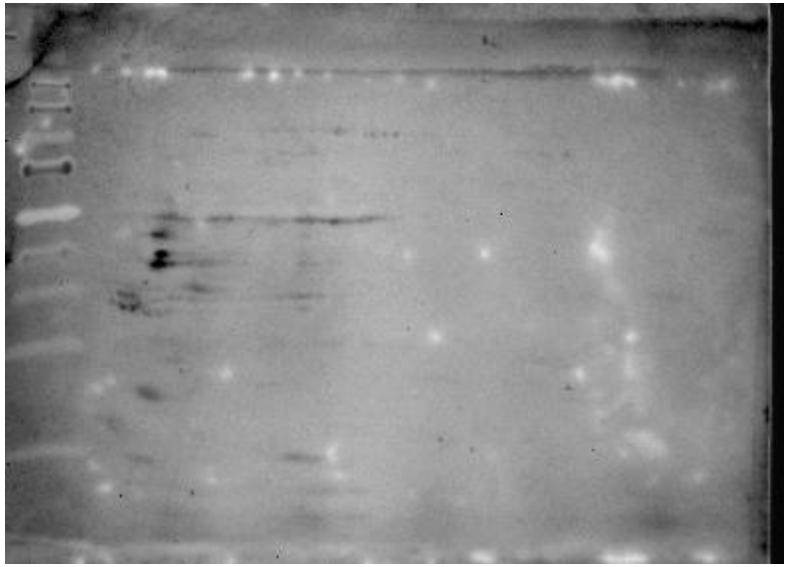

2D- Gel of 22Rv1

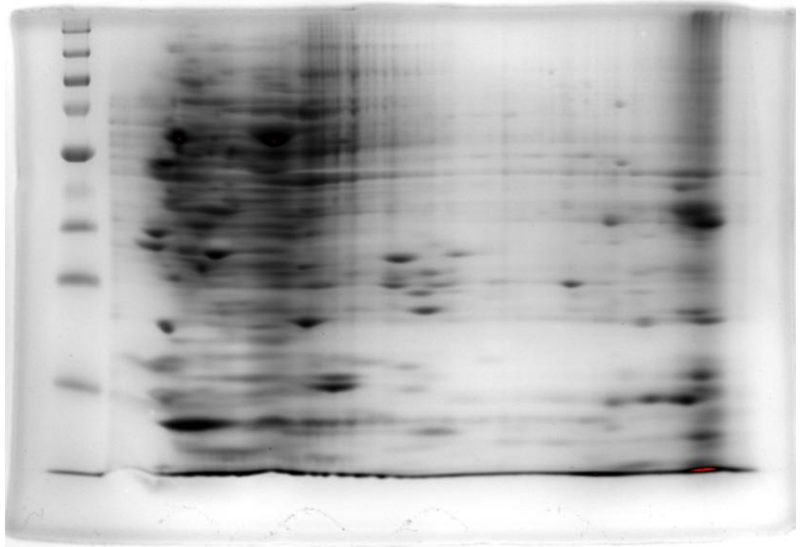

PC-3

Immunoblotting of sera

PCa sera

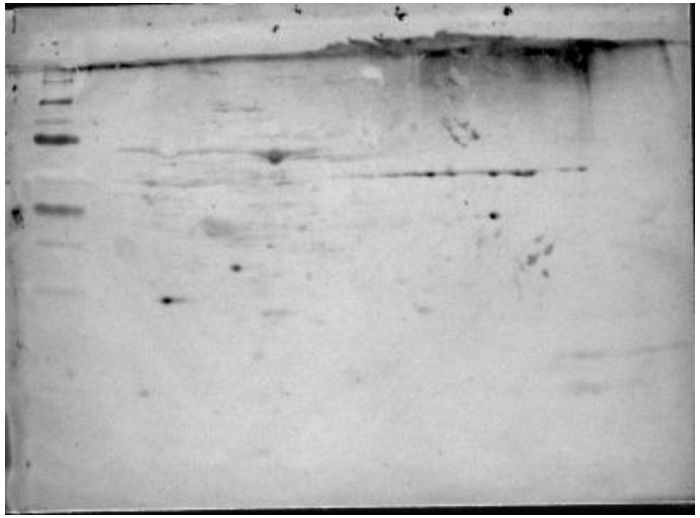

NC sera

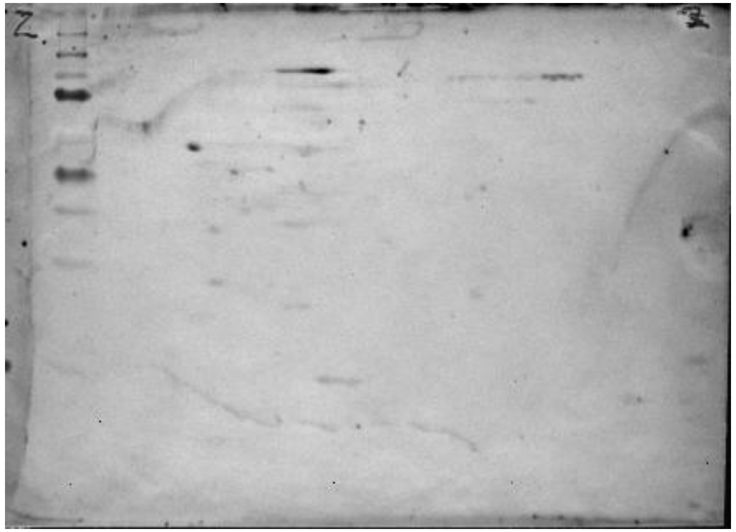

2D- Gel of PC-3

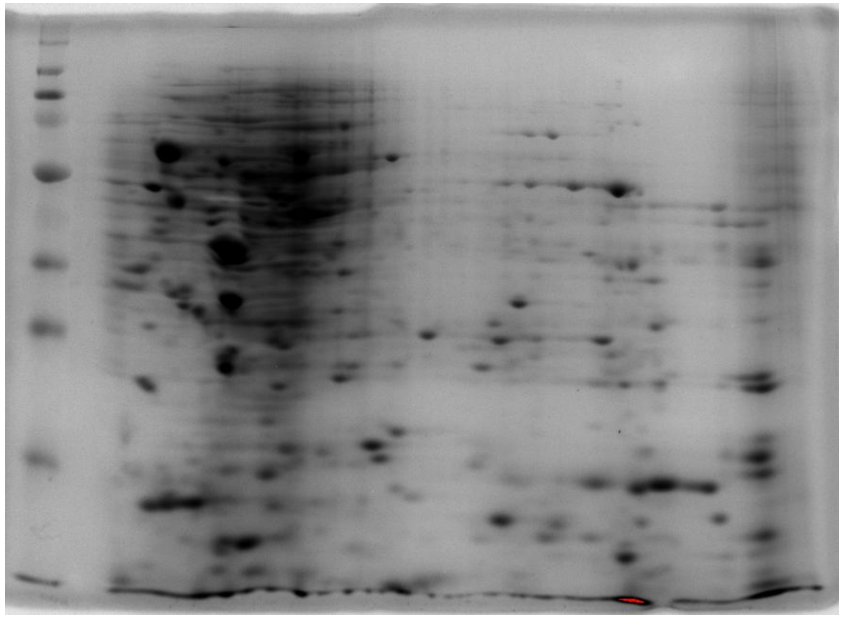

SDS gel of recombinant proteins

Gel 1

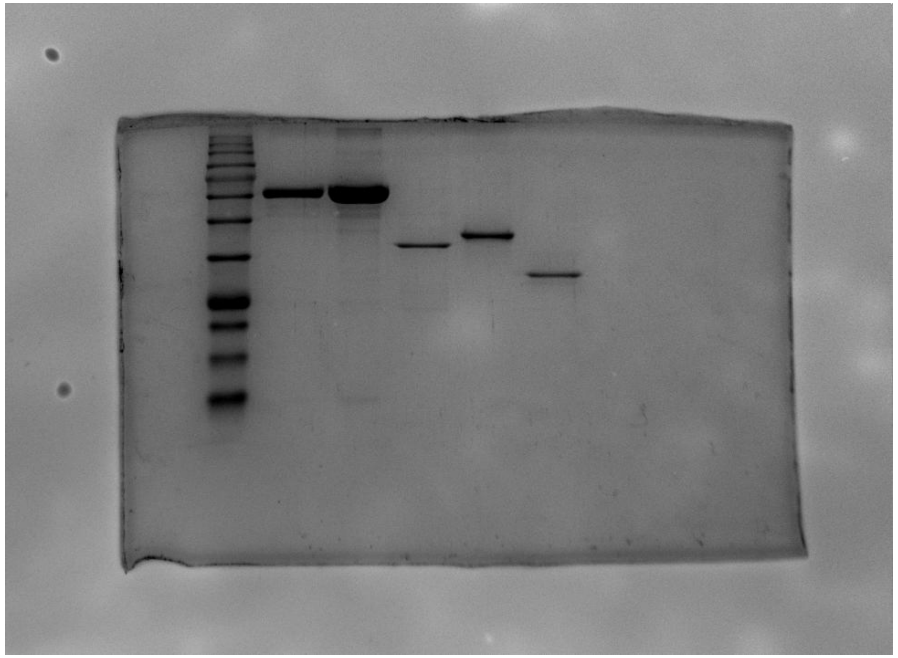

Gel 2

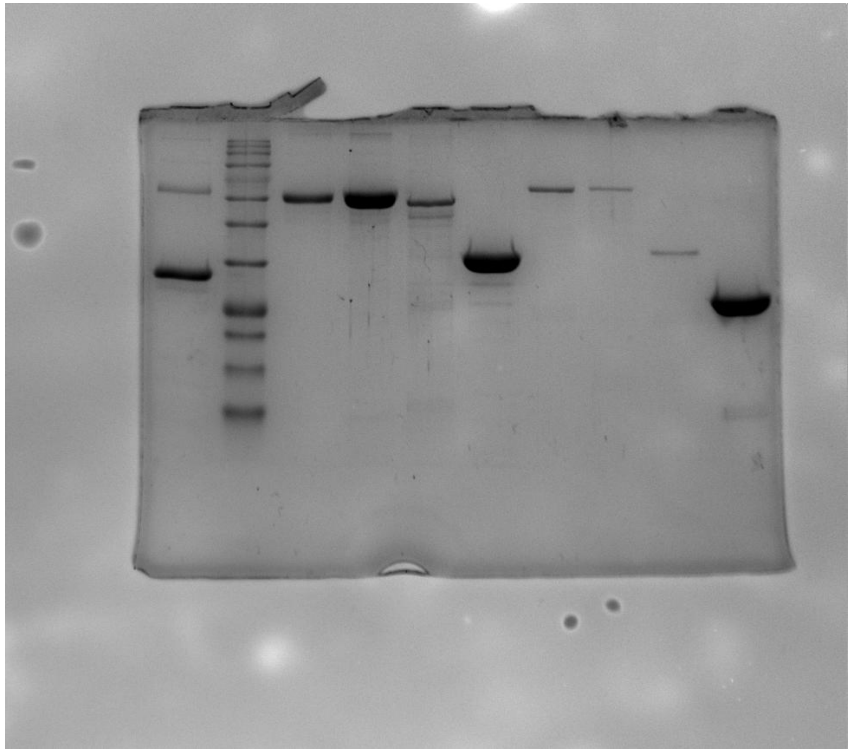

SDS gel of recombinant proteins

Gel 3

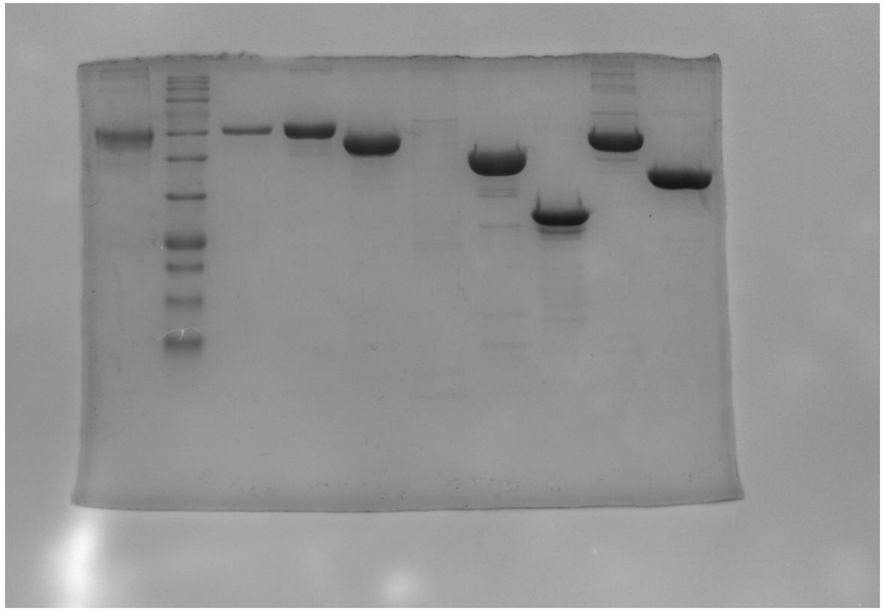

Gel 4

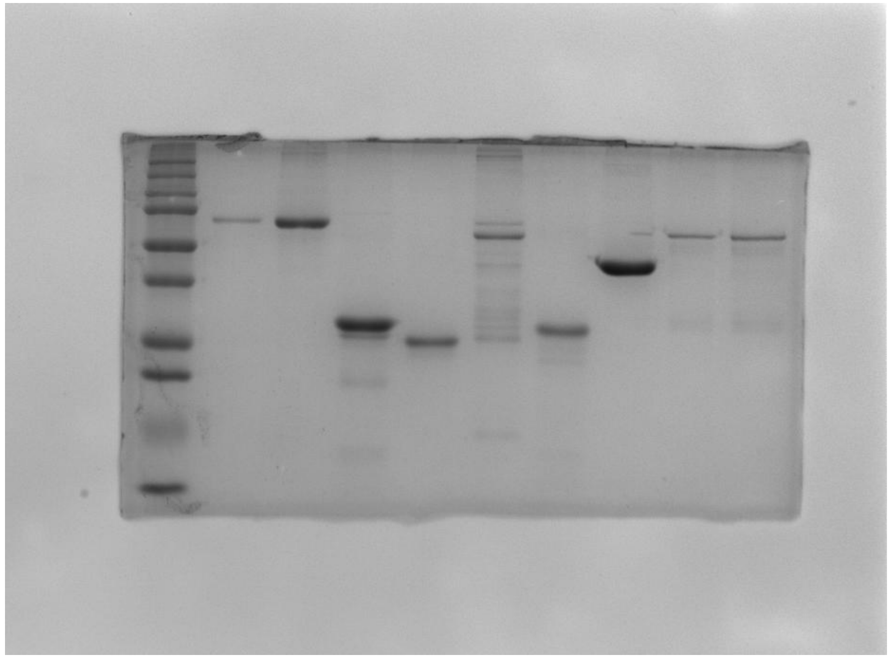

SDS gel of recombinant proteins

Gel 5

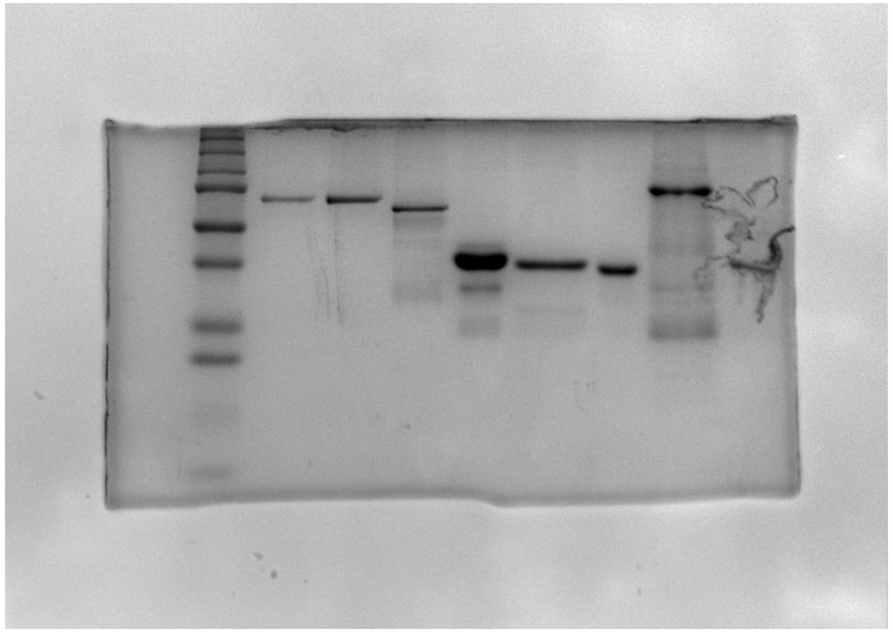

SDS gel of recombinant proteins

Figure S2

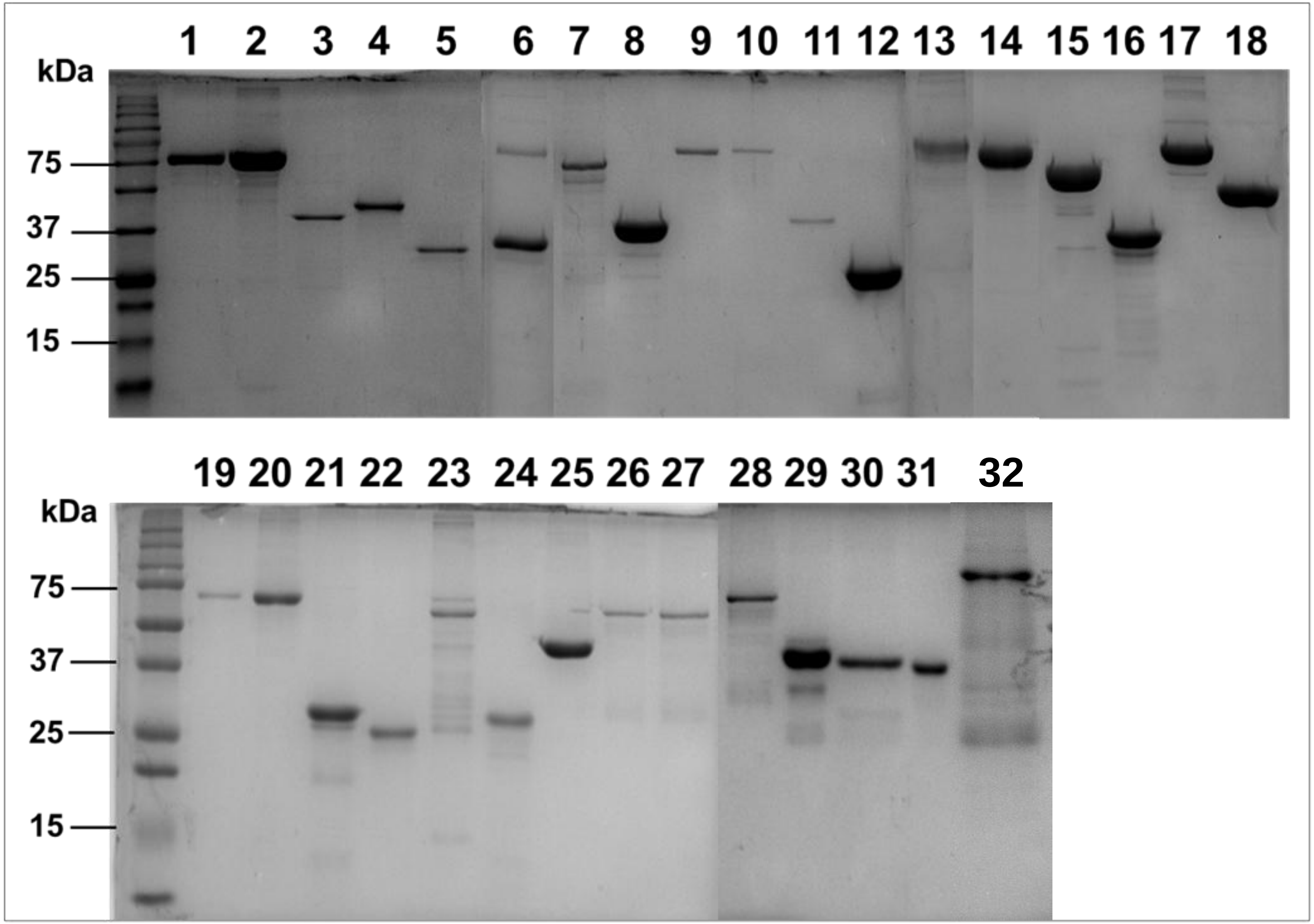

Supplement: Supplementary file 2 — Original gel and immunoblotting [file 41416_2025_3242_MOESM2_ESM.pdf]
